# A Simulation-Based Approach to Aid Development of Software-Based Hardware Failure Detection and Mitigation Algorithms of a Mobile Robot System

**DOI:** 10.3390/s22134665

**Published:** 2022-06-21

**Authors:** Jacopo Sini, Andrea Passarino, Stefano Bonicelli, Massimo Violante

**Affiliations:** 1 Department of Computer and Control Engineering, Politecnico di Torino, 10129 Turin, Italy; massimo.violante@polito.it; 2 INTECS SOLUTIONS S.p.A., 00131 Roma, Italy; andrea.passarino@intecs.it; 3 Department of Electronical Engineering, Politecnico di Torino, 10129 Turin, Italy; s265917@studenti.polito.it

**Keywords:** mobile robotics, real-time and embedded systems, software and system safety, reliability, availability, and serviceability, model development

## Abstract

Mechatronic systems, like mobile robots, are fairly complex. They are composed of electromechanical actuation components and sensing elements supervised by microcontrollers running complex embedded software. This paper proposes a novel approach to aid mobile robotics developers in adopting a rigorous development process to design and verify the robot’s detection and mitigation capabilities against random hardware failures affecting its sensors or actuators. Unfortunately, assessing the interactions between the various safety/mission-critical subsystem is quite complex. The failure mode effect analysis (FMEA) alongside an analysis of the failure detection capabilities (FMEDA) are the state-of-the-art methodologies for performing such an analysis. Various guidelines are available, and the authors decided to follow the one released by AIAG&VDA in June 2019. Since the robot’s behavior is based on embedded software, the FMEA has been integrated with the hardware/software interaction analysis described in the ECSS-Q-ST-30-02C manual. The core of this proposal is to show how a simulation-based approach, where the mechanical and electrical/electronic components are simulated alongside the embedded software, can effectively support FMEA. As a benchmark application, we considered the mobility system of a proof-of-concept assistance rover for Mars exploration designed by the D.I.A.N.A. student team at Politecnico di Torino. Thanks to the adopted approach, we described how to develop the detection and mitigation strategies and how to determine their effectiveness, with a particular focus on those affecting the sensors.

## 1. Introduction

Developing a complex mechatronic system, such as mobile robots, is challenging. They comprise mechanical, electromechanical, and electronic actuation components and sensors. Even if each of these has been appropriately developed following state-of-the-art practices, hence avoiding design defects, random hardware faults (RHF) involving both electronic and mechanical components are unavoidable. These faults can lead to behavioral errors, then to robot-level failures. Moreover, the actuation/sensing hardware is supervised by microcontrollers running embedded software [1]. Although the interactions between the various hardware components in fault-free operations have been analyzed and validated, when a mission or safety-critical system is considered, designers must also cope with the problem of understanding what could happen if a component fails during system operations and identify the best strategies to detect faults and to mitigate their effects. These activities usually are organized in three distinct phases: fault detection, isolation, and recovery (FDIR). In fault detection, the deviation from the intended functionality is recognized. Several techniques are available for this purpose, depending on the failing component. For example, detection mechanisms can use range and plausibility checks considering failures affecting the sensors. Following the detection, actions to isolate the failing components are initiated to avoid further propagating of the fault into an error, then a failure. Finally, recovery actions are undertaken to compensate for its effects on the affected subsystem or bring it into a safe state.

During the concept phase, failure effect analysis is fundamental to guide the development process. In this paper, we propose a simulation-based approach to perform this analysis. As described in the following, on the one hand, it allows assessing the possible deviations from the intended functionalities and the extent of such deviations. At the same time, on the other hand, it simplifies the validation of the implemented fault detection, isolation, and recovery mechanisms.

This proposal allows the various subsystems (electrical, electronic, mechanical) to be simulated alongside the embedded software, offering information on the hardware/software behavior. Moreover, RHFs can be injected into the hardware components, thus analyzing how their effects propagate through various subsystems.

To benchmark the proposed approach, an experimental assistance mobile robot (rover), designed to aid astronauts on Mars, has been analyzed from both mechanical and electrical failure points of view.

To perform the analysis, we choose to follow two industrial standards:

- The ECSS-Q-ST-30-02C [2], for what regards the hardware-software interaction analysis (HSIA);
- The novel failure mode and effect analysis (FMEA) manual released in June 2019 from the AIAG and VDA [3].

The contributions of the approach presented in this paper are three-fold.

In the first place, it improves the objectivity and repeatability of the FMEA and hazard analysis and risk assessment (HARA) phases by a simulation-based approach.

Moreover, it describes how to continuously verify from the early development phases, as further subsystems are implemented step by step to the final design if they fulfill their mission and safety requirements.

Finally, it proposes a way to simplify the safety assessment of the hardware/software interactions, especially in those cases where mechanical, electrical/electronic components, and embedded software are involved in determining complex behaviors of the entire robot. This paper aims to propose an approach to guarantee functional safety and mission requirements: aspects regarding tolerance to errors caused by space radiations are not considered in the scope of this work, under the hypothesis that the chosen computation platform is radiation hardened.

The rest of the paper is organized as follows. Section 2 describes the state of the art in mechatronic systems design, the role of the embedded software, and the main concepts about functional safety, HARA, FMEA/FMEDA, fault injection, simulation environments, and, finally, the background of the proposal. Section 3 describes the proposed approach, while Section 4 describes the experimental setup, composed of models of the rover itself (virtual rover) and models of the subsystems involved in the analysis, and summarizes the simulation environment. Section 5 describes the improvement measures (actions taken) in terms of detection and mitigation algorithms. Section 6 discusses their effect and efficacy on the virtual rover. Section 7 draws conclusions on this paper.

## 2. State of the Art

The proposed methodology combines three well-established techniques: fault injection, failure mode, effect, and diagnostic analysis (FMEDA), alongside physical simulation.

The following discussion about the state of the art has been divided into five macro-areas:

- Functional safety;
- HARA and FMEA;
- FMEDA and fault injection;
- The simulation environment;
- The background of the proposed approach.

### 2.1. Functional Safety

We can summarize the concept of functional safety as the ability of a cyber-physical system to react on time and adequately to the external environment. If misbehaviors of the system can damage people’s health or the environment, we are in the presence of a safety-critical system. If system misbehavior can lead to a significant amount of money loss, we are in the presence of a mission-critical system. For the development of safety-critical systems, many standards have been defined. The most important is the IEC 61508 [4], which is the generic one. Starting from this standard, newer ones have been developed, like the ISO26262 [5] (automotive industry), EN50126 [6] (railway industry), and the DO-178C [7] (software embedded into avionics). Even if they are applied to different fields, they share a strict development process where:

- The system dependability has to be taken into account in all the phases;
- A hazard analysis and risk assessment (HARA) is needed to formalize the requirements;
- An FMEA is required to be performed;
- It is necessary to assess if the design is able to achieve all the requirements found during the HARA and FMEA.

The hardware and software design also involves a phase where the system’s fault detection, mitigation, and isolation capabilities have to be taken into account through FMEDA. We choose to describe the concept of functional safety, even if the benchmark application is mission-critical, primarily since we claim that in this way, the proposal can interest a wider audience of scholars.

### 2.2. FMEA and HARA

FMEA and HARA have some weak points [8,9,10]:

- FMEA can take into consideration only one failure mode (a component can fail in different manners for different causes) at a time, so it cannot assess the effect of correlated or contemporary faults;
- There is a lack in objectivity, in particular when FDIR mechanisms are implemented thanks to the embedded software;
- There is a lack in repeatability;
- They are time-consuming activities.

The peculiarity of FMEA causes the first issue since the number of combinations increases exponentially with the number of multiple-point failures (i.e., those failures not dangerous by themselves but that are dangerous in combination with others) to be considered but, thanks to the simulation-based approach, it is possible to:

- Improve the repeatability thanks to model and workload definitions. These allow repeating the simulations in a deterministic way (with the only limitation on the quality of the adopted models). This aspect is an improvement with regards to the proposals introduced by [8,9], where classification tables aid the HARA. In this case, classification tables are compared against the simulation results and not with the estimation of the human experts, in a similar way as it has been performed in [10];
- Improve the objectivity thanks to the simulation results [10];
- Speed up the analysis: simulations are faster with respect to a handmade analysis. Concerning the models, it is possible to re-use those developed to design the control systems in nominal conditions. Moreover, if it is possible to describe (as in this case) the fault models only at the behavioral level, they are simple to develop.

### 2.3. FMEDA and Fault Injection

Fault injection techniques [11,12,13,14] have been adopted in recent years in all industrial fields. There are many proposals in the literature about this topic with two different goals: to improve the reliability of the product under design and perform the validation process.

Usually, during the FMEDA, the failure mode effect classifications are performed manually by skilled designers. Due to the increasing complexity of mechatronic systems, taking into account the hardware redundancies, embedded software mitigation, and detection capabilities is becoming more difficult every day. For the sake of this work, we are interested in how to inject failures inside a simulation. As described in [11], we need a:

- **Fault injector** (or **saboteur**) to inject faults into the target system;
- **Fault library**, a collection of semi-formal descriptions, suitable for the chosen simulation environment of those failure modes we are interested in;
- **Mission database** to describe the specific tasks we want to complete with the system when used for the purposes for which it has been designed for;
- **Controller** to coordinate all the set-up components;
- **Monitor** to log all events happening during the injection;
- **Data collector** to perform data collection;
- **Data analyzer** to perform data processing and analysis.

The following will describe how safety engineers can map these components to set up a fully simulated fault injection environment. How to adapt such a fault injection system to perform HARA is described in [10], while [15] describes how to adapt it for performing FMEDA.

In the rest of the paper, instead of failure (or faults, when it does not provoke confusion), the acronym failure mode (FM) is used since a component can fail differently.

During the FMEDA, the failure mode effect classification is done, considering if it can be detected and if it can lead to a violation of safety (or mission) requirements.

### 2.4. Simulation Environment

A digital closed-loop control system is usually composed of the following parts:

- The **physical process** to be controlled. In this case, this refers to the mobility system of the rover itself;
- **Sensors**. In this case, this refers to the absolute encoders mounted on the hinges of the rocker-bogie and the steering systems for the external wheels, and the relative encoders on the hubs of the wheels;
- **An analog electronics input conditioning stage** (to adapt sensos outputs to processing state inputs);
- A processing stage (usually a microcontroller);
- **An analog electronics output conditioning stage** (to adapt processing stage outputs to actuator inputs);
- **Actuators** and their accessories (in this case the reduction gears).

The simulation environment is in charge of simulating the rover mechanics, interacting with the terrain, the sensors, and actuators, and running the embedded software inside the simulation environment. Since we used commercial off-the-shelf (COTS) encoders with integrated conditioning stages, we decided to simulate them at the behavioral level [16]. How to set up the simulation system with fault injection is described in [15], while the FM effect propagation up to the system level (in the case of the cited paper, a vehicle) is reported in [17].

### 2.5. Background of the Proposed Approach

The proposed approach has been obtained by combining the AIAG&VDA [3] and the ECSS-Q-ST-30-02C [2] manuals alongside the simulation system proposed in [17]. The manual [3] describes a state-of-the-art FMEA process, while the [2] has been followed for what regards the HSIA since this topic is not addressed explicitly by [3]. While the manual [2] was released in its last version in March 2009, ref. [3] is a novel one since it was released in June 2019.

The core of the approach is the process described in the AIAG&VDA manual [3]. It is divided into seven steps. The first three are about system analysis, specifically planning and preparation (1), structure analysis (2), and function analysis (3). The other three steps regard failure analysis and risk mitigation and are failure analysis (4), risk analysis (5), and optimization (6). The last concerns risk communication (7) through results and safety-related documentation.

We start with the design FMEA process described by steps 2 to 5 of [3]. Phases 2 and 3 are performed by hand by following the manual. Starting from phase 4, we found out that there are two options: it is possible to complete it by hand as usual for those FMs with clear effects at the system level or by a simulation-based approach without trivial FM effects. Thanks to the simulator, it is possible to analyze the effects of the FMs we want to assess by injecting them individually.

The AIAG&VDA manual [3] requires, for each FM, for its score to be defined in terms of three different parameters: severity, occurrence, and detectability. Each of these three ranges from 1 to 10.

The severity measures the worst possible consequence when the FM occurs. This parameter is scored from 1, which indicates that the FM has negligible consequences on the system, up to 8, where a primary function is lost. A score of 9 indicates that the FM provokes non-compliance with regulations, while 10 means that there can be consequences on people’s health or the environment. Therefore, only those FMs with a severity score of 10 are, strictly speaking, safety-critical. Thanks to simulation, it is possible to obtain valuable results to determine this parameter.

The occurrence is determined by hand based on statistical evidence, so the simulation cannot help to decide this parameter.

The detectability is a metric about the capacity of the system to detect its possible FMs. In this parameter, we have the crucial advance. Thanks to the simulation, it is possible to observe if the embedded software can trigger the most appropriate mitigation algorithm (SW action [2] terminology), as described in the following of this paper. Moreover, it is possible to obtain valuable data on the effectiveness of the developed mitigation algorithms. Combining these three parameters, obtained in phase 5, results in an **action priority** (AP) that can assume three levels: low (L), medium (M), and high (H).

The FMs with an AP higher than L need to be optimized through FDIR mechanisms during phase 6.

The simulation environment is based on the one initially proposed in the papers [15,16,17], which feature the fault injection methodology proposed in [11]. This proposal merges the characteristics of the last two papers. In [17], it is proposed how to propagate the FM effect at the whole system level (in the case of the cited paper, wherein an automotive scenario is considered, the system is a sedan car), while in [16], behavioral models are adopted for the fault-free subsystems. With respect to [17], this methodology also allows injecting faults in the mechanical components, while concerning [16], the novelty is to inject faults thanks to behavioral models. Both [16,17] run the embedded software (and, in particular, the software-implemented FDIR mechanisms) alongside the physical simulator: this characteristic is also present in the proposal of this paper.

## 3. Proposed Approach

This section describes the proposed approach.

The description is split into 3 parts:

- FDIR performance assessment;
- The simulation environment;
- A description of the fault models adopted for the FMs affecting the components of the benchmark application.

### 3.1. FDIR Performances Assessment

For the sake of this work, we have not performed steps 1 and 7 of the AIAG&VDA manual. Step 5 is briefly described, while we focus on phase 6 since our goal is to assess the software capabilities to detect, isolate and mitigate the FMs against the requirements determined in phase 5. The HSIA from [2] plays its roles between the phases risk analysis (5) and optimization (6), where we have to determine the severity and the detection capabilities for the considered FMs, respectively.

#### 3.1.1. Phase 5 AIAG&VDA

During phase 5, it is not possible to assess the FDIR mechanisms of the design since the manual requires not considering advanced detection mechanisms like the one based on the embedded software, which is of interest for this paper. To comply with this requirement, we run the first bench of simulations during this phase, one for each one of the considered FMs. In any case, other than to decide the severity parameter required by the manual, the simulation results obtained in fault-free conditions are needed to get the system’s expected nominal behavior.

This phase is crucial to determine which FDIR mechanisms to implement into the design.

#### 3.1.2. HSIA (ECSS-Q-ST-30-02C) and Phase 6 (AIAG&VDA)

**Figure 1:**
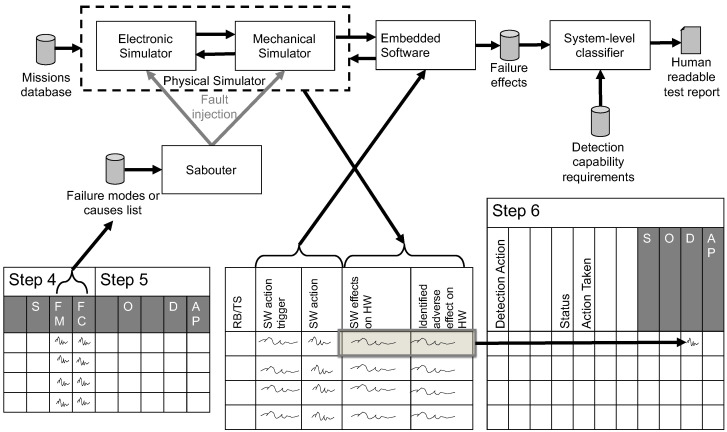
Proposed methodology flow-chart. Tables at both sides follow the structure indicated in [3], while the central one follows the HSIA described in [2]. List of abbreviations from the left to right: severity (S), failure mode (FM), failure cause (FC), detectability (D), occurrency (O), action priority (AP).

After a first draft of the needed FDIR mechanisms has been developed, it is possible to start the optimization phase (6) of the AIAG&VDA process. Now, the considered system is simulated as in phase 5, including the newly developed FDIR mechanisms. These simulations can be repeated every time they are needed, allowing an iterative approach to design these mechanisms until their required detection and mitigation specifications have been achieved. In the terminology of the HSIA, as specified in [2], the requirements for our FDIR mechanisms implemented by the embedded software are the ones described in the triggers and perform the actions specified in the “SW action trigger” and “SW action” columns of the central table shown in the Figure 1.

A SW action can be:

- An emergency procedure to move the system into a safe state (fail-safe behavior);
- An action to isolate the fault, avoiding its propagation to the rest of the system;
- An algorithm to mitigate the FM effects (fail-operational behaviors).

Thanks to the simulation results, it is possible to assess the effect of the SW on the HW (the purpose of the HSIA). A properly developed FDIR implemented by software aims to improve the reliability of the design, but unfortunately, it can also add negative side effects that can unexpectedly affect the HW, for example, causing an increase in vibrations. Once the SW effects on HW have been obtained, we can finally assess the detection and mitigation capabilities of the embedded software as required by phase 6, allowing us to determine if the pros/cons balance of the proposed algorithms is positive.

### 3.2. Simulation System

The proposed simulation system is composed of (see Figure 1):

- A **saboteur** that modifies both the circuit and the mechanical models to simulate the FM we want to inject;
- A **missions database**, which is a file containing the descriptions of the scenario and operative conditions in which we want to assess the effects of the FMs;
- **Electronic and mechanical simulators**, which are in charge of performing the SPICE-level simulation of the analog conditioning system and the mechanical simulation of the rover, respectively. An interoperability layer is provided to put in communication with the two domain-specific simulators;
- A **failure modes or causes list**, which is a file containing the list of the FMs for each of the component classes of the considered system and a set of instructions, interpretable by the saboteurs, on how to inject them into the simulation environment;
- **Embedded software**, the code that will implement the detection and mitigation strategies in the considered system;
- A **controller**, the function that manages the fault injector and the circuit simulator;
- A **system-level classifier**, which applies the classification rules to assess the FM effect in terms of severity. Adding redundant components makes it possible to spread the severity level of the missing or not correctly done functions on different subsystems. The approach verifies that the redundant modules work properly in failure isolation and recovery (mitigation). It can also trace the detection performed by the embedded software, showing when the detection algorithm works appropriately or when a false positive (detection of an FM that does not exist) or false negative (an actual FM is not recognized) occurs.

The joint usage of electronic and mechanical simulators composes the physical simulator. The controller (not shown in Figure 1) keeps the information about the injected fault and sets the mission on both simulators. The system is firstly simulated in fault-free (golden) conditions to determine its expected behavior when dealing with each of the workloads contained in the library. These outcomes are stored in the fault-free simulation results database.

Once golden simulations have been performed, it is possible to start with the fault-affected simulation. In this way, obtaining the item-level behaviors in case of failures without (phase 5) and with (phase 6) mitigation algorithms is possible.

From these components, it is possible to describe how they are integrated to obtain a suitable simulation environment, as shown in Figure 1. This figure shows how the listed components interact together and how they allow the shown tables to be filled. The left table is from the AIAG&VDA Manual, which is the result of phases 4 and 5, so its content is an input for this process. The center table is from the ECSS-Q-ST-30-02C standard and describes the hardware-software interaction analysis (HSIA). In this table, the columns “SW action trigger” and “SW action” are, in our methodology, the embedded software we developed with a proper interface to interact with the simulation system. The columns “SW effects on HW and “Identified adverse effect on HW“ are filled from the simulation results, automatically from a set of rules if there are quantitative parameters from the simulation or by hand if the analysis is only qualitative. In this paper, we adopted only qualitative classification rules, as will be explained in Section 3.4.

The table on the right is again from the AIAG&VDA manual, and it is the result of phase 6. From the manual point of view, we formally obtained the system’s detection capabilities, and information about the new severity obtained thanks to the mitigation. From the practical point of view, it is possible to again assess the severity obtained from phase 4 during phase 6, but if it is a good practice or not is still an open discussion, we do not want to focus on this paper.

### 3.3. Fault Models

Faults can be simulated by two different paradigms [18]:

- Model-based (structural or parametric), if implemented by modifying the physical model (structural) or the parameters (parametric) on which the component relies to perform its functionality;
- Signal-based (behavioral), if the injection is done by changing a signal produced by the affected component.

Due to the complexity of the rover, composed of various subsystems, and our focus on the embedded software detection and mitigation capabilities, we used model-based (structural) paradigms to inject failures of actuators and signal-based (behavioral) models for the sensors.

At the end of each fault-affected simulation, the classifier applies the classification rules to determine the severity of the considered FM. For some faults with only a qualitative description, the classification is performed by hand.

To inject faults corresponding to the various considered FMs in the simulations, we developed fault models to represent them with sufficient detail. We will describe them for each class of components.

#### 3.3.1. Sensors

- Absolute encoders: when one of them fails, it provides a position readout equal to its zero position (0 deg in the simulation).
- Relative encoders: when one of them fails, it provides a speed readout equal to zero (hence 0 rpm).
- Hall effect speed sensors: similar to the relative encoders, when one of them fails, it provides a random speed readout.

#### 3.3.2. Passive Components

- Reduction gears: when one of them fails, it remains stuck, preventing the affected wheel from rotating.

#### 3.3.3. Actuators

- Clutches: the considered ones are normally open. They are monostable, so when their solenoid is operated, they transfer torque from the motor to the wheel. When one fails, the traction control can no longer engage it, so the traction motor cannot provide torque to the affected wheel.
- Traction motors: when one of them fails, it remains stuck. Due to the presence of the reduction gear, the affected wheel remains stuck. Due to this effect, the need to install a clutch between the traction motor and its reduction gear.
- Steering motors: when one of them fails, it remains stuck. Due to the presence of the reduction gear, it is not possible to move the wheel, which remains with the relative angle applied by the motor the last time it was able to operate.

### 3.4. Classification Rules

Classification rules are needed to determine if the implemented detection and mitigation strategies are effective.

Regarding detection, the software changes its states or at least a variable to communicate to the other subsystems that a failure has been detected. Important information to be reported is the time elapsed from the injection of the fault to its detection.

Regarding the mitigation strategies, the classification rules can be of two types:

- A comparison between fault-free and fault-affected simulation outcomes;
- Specification-based, where the expected behavior can be compared with the expected one reported on the item specifications.

The classification rules considered in this paper are all specification-based.

## 4. Experimental Setup

The considered benchmark application is a mobile robot. More in detail, it is a Mars-exploration rover designed by the D.I.A.N.A. student team of Politecnico di Torino. We chose such a system since it is possible to access all the mechanical drawings, electronic schematics, and the embedded software in charge of moving the robot on the Mars surface. This kind of access is impossible due to intellectual protection issues in an industrial scenario.

### 4.1. Mobility System

The mobility system is built on a rocker-bogie suspension system that allows the rover to climb over obstacles up to twice the diameter of the wheels without springs [19].

It involves 5 joints: 2 between the rockers and bogies, 2 between the rockers and the fixed frame, and 1 in the middle of the torsion bar.

The fixed frame contains scientific instrumentation, control computers, and supports an arm. The rover has 6 traction wheels, each with an independent permanent magnet motor and a reduction gear. Only the 4 wheels at the extremities (both front and rear) can steer, thanks to 4 independent stepper motors and reduction gears.

**Figure 2:**
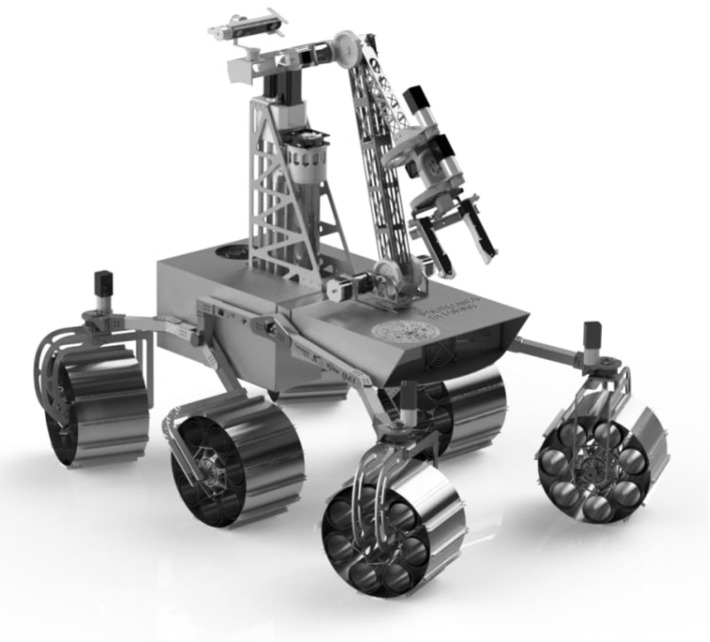
A 3D rendering of the Ardito Rover developed by the D.I.A.N.A. student team of Politecnico di Torino.

A render of the rover is shown in Figure 2.

Its control system is comprised of a central control unit and 6 peripheral control units, one for each of the 6 wheels.

Due to this mechanical design, this system is managed by complex software in charge of making it simple to use for astronauts and capable of operating autonomously.

Sensors are installed to measure angles and angular velocities. There are absolute encoders to measure angles of the steering wheels with respect to the frame and relative encoders to measure angular velocities of the wheels. The encoders on the motors (regardless of whether they perform an absolute or a relative measure) have been installed downstream and upstream of the reduction gears. Moreover, the rocker-bogie joints positions are measured by 3 absolute encoders: 2 of them inside the joints between the rockers and bogies structure and the remaining one on the torsion bar.

Since no mechanical link between the wheels is present, the correct configuration of the steering system is guaranteed, thanks to the absolute encoders readouts, only by the mobility embedded software [20]. This configuration is calculated using an Ackermann model.

Even if the rocker-bogie structure is the most suitable for our purposes, it implies that there are no mechanical differential gears to move the wheels in a way that allows the rover to move smoothly, even in the simplest case where it has to follow a straight line, without a complex strategy. Hence, the need to design a microcontroller-based mobility system capable of harmoniously moving the traction and steering motors allows the system to be guided in manual mode, via a stick controller, and through autonomous driving algorithms.

We benchmark the approach on the mobility subsystem of this rover, focusing on studying how the embedded software can detect and mitigate some of the possible FMs, considering the affected components, the encoders, the motors, and the clutches between the traction motors and the reduction gears. In this case, the main goal is to reduce the damage to the rover caused by the possible RHFs. However, since it is designed to aid astronauts in a hostile environment, we prefer fail-operational behaviors for some of the faults when we find a good trade-off between the possible damage of the rover itself and simplification of the operation for the astronauts.

As the first step, the mechanical components have been designed. Once the mechanical drawings were ready, we performed an extensive handmade failure mode and effect analysis (FMEA) following phase 5 of the novel AIAG&VDA manual [3]. We helped ourselves to assess their effects for some of the considered faults thanks to the simulation system.

Since computer-aided design (CAD) tools are adopted to design all the mechanical components, the mechanical drawings are also used to perform simulations with reasonable accuracy.

These are usually needed to design the control algorithms and, in particular, to check if the designed controller can reach the required nominal performances [21]. Extending the simulation-based approach to this new purpose reduces the cost and development time.

From the mechanical point of view, we considered the frame of the rover itself, the rocker-bogie suspension system, and of course, the 6 wheels. The 4 outer wheels can steer. Since the suspension system has no shock absorption system, the wheels are designed to be elastic to dampen the roughness of the ground.

The mobility system is composed of these 55 components:

- Six traction brushless direct current (BLDC) motors (TM);
- Six traction reduction gears (TRG);
- Six clutches between the traction motors (TC);
- Four steering stepper motors (SM);
- Four reduction gears for the steering motors (SGR);
- Six relative encoders installed on the traction motors (speed of the motor) (E-TM);
- Six relative encoders installed on the traction reduction gears (speed of the wheel) (E-TRG);
- Six hall effect speed sensors installed on the wheels (H-TRG);
- Three absolute encoders, 2 installed on both rocker-bogie hinges, the other on the torsion bar one;
- Four absolute encoders installed on the steering motors (position of the steering motor) (E-SM);
- Four absolute encoders installed on the steering after the reduction gears (position of the wheel) (E-SGR).

### 4.2. Setup of the Simulation Environment

We wanted to set up a simulation environment to test our software without needing the physical rover. We searched for a suitable environment, finding the best solution in the combined usage of CoppeliaSim [22] alongside MathWorks MATLAB/Simulink [23].

CoppeliaSim was in charge of simulating the multibody (mechanical) components, and the interaction between the wheels and the terrain [24], while Simulink simulated the sensors and electronics.

The control and FDIR software have been developed by Model-Based Software Design, resorting to Simulink and generating the code in C language by MathWorks Embedded Coder (Simulink Coder). It simplifies the integration between the environments: the algorithms can control the simulation thanks to the C++ APIs exposed by CoppeliaSim. The Message Queue Telemetry Transport (MQTT) protocol has been adopted since it is used in the real rover to communicate with the base station.

#### 4.2.1. Fault Injection

We implemented the ability to inject faults in the mobility system to test the controller response and assess reliability, thanks to the simulations. Different faults have been implemented simulating their effect on the rover, as described in Section 5.

#### 4.2.2. Data Logging

The mobility control software generates a comma separated values (CSV) file in order to keep track of all the signals involved. This feature helps debug and to check the effectiveness of mitigations over the FM effects.

**Figure 3:**
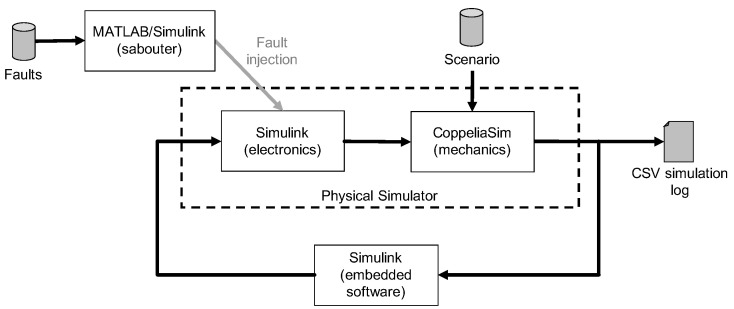
Scheme of the implemented simulation environment, with indication of the chosen software.

Figure 3 shows the elements involved in the simulation. We want to highlight the central role of the embedded software in achieving the expected results from the simulations.

## 5. Improvement Measures

This section describes all the activities performed to improve the reliability of the rover. In particular, Section 5.1 summarizes the results of the FMEA analysis performed on the mobility system of the rover, while the following subsections describe the implemented detection and mitigation algorithms, respectively, for the traction and the steering subsystems.

### 5.1. Summary of the FMEA

A complete discussion of the FMEA is out of the scope of this paper; hence, we reported only the needed information to make it possible for the reader to understand the required FDIR performances.

**Figure 4:**
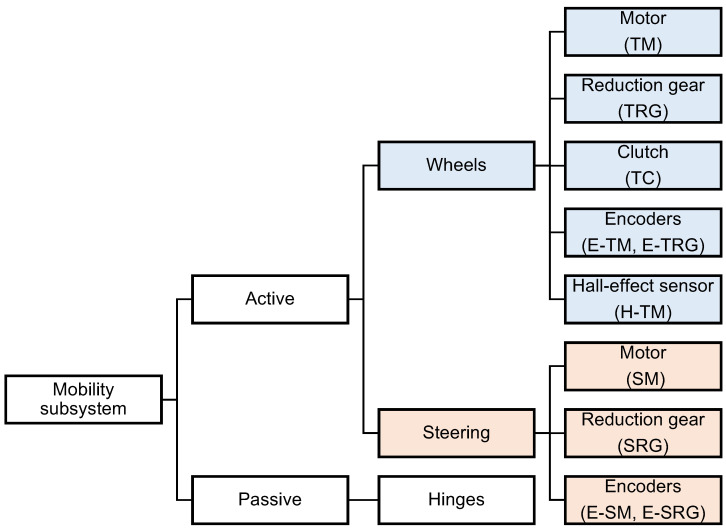
Mobility system hierarchical organisation.

As already said, the mobility system is composed of 55 components; hence, we have $2^{55}$ possible FMscombinations (each component can fail only in one way, so it is associated with only one FM). Of course, such complexity is not manageable, so we simplified the problem by considering its sub-domains. We divided the detection algorithm following the hierarchy shown in Figure 4.

Regarding the active part, we divided it into six sub-domains, with each one representing a wheel and the relative steering system. From the conceptual point of view, we managed the center wheels, without steering motors, as subsystems where steering failures are impossible. This simplifies the algorithm since, in this way, the detection mechanism can be replicated 6 times, on each one of the ECUs in charge of a wheel, without the need to keep two different versions. For the sake of this work, we decided to not perform checks on the encoders on the rocker-bogie joints (the two hinges and the bearing on the torsion bar) since, on the one hand, their relative position depends on the terrain and, on the other hand, there are no redundancies of these measurements, so it is difficult to check onboard the rover if they are working properly [25]. In any case, these encoders are installed in a better position than those installed on wheels and steering, lowering the probability that one of them would become damaged during the operations.

Regarding the active parts, we can find that, for each wheel, there are up to 10 components: 3 actuators (TM, TC, and SM), 2 passive elements (TRG and SRG), and 5 sensors (H-TM, E-TM, E-SM, E-TRG, E-SGR). The two central wheels cannot steer, so they do not have SM, SRG, E-SM, and E-SRG. The reader can find more details about these items in Section 4.1.

From the $2^{10}$ (1024) possible combinations of the 10 components’ FMs, we defined 13 failures groups, described in Table 1. For each one of them, we indicated the involved component and the expected action at the rover level.

### 5.2. Taken Actions

To improve the reliability of the mobility system of the rover, we developed detection algorithms able to use measures from encoders and hall sensors to detect failures on a single wheel (in other words, to implement the algorithms described in the previous subsection). These are needed to trigger mitigation strategies.

#### 5.2.1. Detection Algorithms (Traction Subsystem)

To define the rover state and trigger mitigation actions, we must determine which of the 13 failure groups described in Section 5.1 represents the detected FM. To determine the affected components, we need detection algorithms. Here, we consider only the failures affecting the traction subsystem (for failures of the steering subsystem, see Section 5.2.6).

#### 5.2.2. First-Level Detection Algorithms (Traction Subsystem)

For this purpose, we developed 6 different first-level detection algorithms to detect non-plausible measurements between couples of the three sensors involved (E-TM, H-TM, E-TRG) and the considered wheel velocity command. There are $C_{4,2}=\frac{4!}{2!\cdot (4-2)!}=6$ possible combinations obtained by considering two sensors at a time (a sensor cannot disagree with itself, and the order does not make any difference). These combinations are shown, in a matrix form, in Table 2. When a check fails, the relative flag is set. The checks are defined as comparing the difference between the two considered measurements and a threshold.

In more detail:

- If both the measurements are 0, it has no sense to perform the comparison;
- If one of the inputs is equal to 0 and the other is not not, their difference is compared with a threshold equal to 2 rpm; the 2 rpm threshold has been chosen to avoid possible divisions by 0;
- If both the inputs are different from 0, their difference, normalized by the first input, is compared with a threshold equal to 0.2 (corresponding to a 20% disparity).

For each one of those algorithms, we indicate the flag name and the two sensors (or commands) involved in the comparison.

- TF1 (E-TM/Wheel velocity command). The flag TF1 is raised when the difference between the wheel velocity command and the velocity measured by the encoder E-TM installed on the motor shaft is greater than the threshold.
- TF2 (H-TM/Wheel velocity command). The flag TF2 is raised when the difference between the wheel velocity command and the velocity measured by the hall sensor H-TM installed inside the motor is greater than the threshold.
- TF3 (E-TM/H-TM). The flag TF3 is raised when the difference between the velocity measured by the encoder E-TM installed on the wheel and the one measured by the hall sensor H-TM installed inside the motor is greater than the threshold.
- TF4 (E-TM/E-TRG). The flag TF4 is raised when the difference between the velocity measured by the encoder E-TM installed on the wheel and the one measured by the encoder E-TRG installed on the reduction gear (divided by the reduction ratio of the gear itself to obtain a comparable value) is greater than the threshold.
- TF5 (H-TM/E-TRG). The flag TF5 is raised when the difference between the velocity measured by the hall sensor H-TM installed on the wheel and the one measured by the encoder E-TRG installed on the reduction gear (divided by the reduction ratio of the gear itself to obtain a comparable value) is greater than the threshold.
- TF6 (E-TRG/Wheel velocity command). The flag TF6 is raised when the difference between the wheel velocity command and the one measured by the encoder E-TRG installed on the reduction gear (divided by the reduction ratio of the gear itself to obtain a comparable value) is greater than the threshold.

Table 2 recaps how the traction flags (TFs) are mapped to generate mitigation triggers.

#### 5.2.3. Second-Level Detection Algorithms (Traction Subsystem)

The first-level detection algorithms generate 6 flags, so we have 64 ($2^{6}$) possible combinations. These second-level ones are needed to group these flags.

There are 7 conditions based on the number of flags that are set:

- All flags cleared: no plausibility problems are detected; hence no failures are affecting the sensors; there is 1 combination of this kind.
- 1 flag set: the state is incoherent but tolerated without triggering any mitigation; there are 6 combinations of this kind.
- 2 flags set: the state is incoherent but tolerated without triggering any mitigation; there are 15 combinations of this kind.
- 3 flags set: 6 of them represent with certainty the failure of a component, while 14 are not sufficient to determine the affected sensors and are hence tolerated without triggering any mitigation; there are 20 combinations of this kind.
- 4 flags set: no one of them is sufficient to represent with certainty the failure of a component, but if one or more subsets of three flags are one of the 6 sufficient to represent a failure, these failures are taken into account. If not, the wheel is considered in the all-flag set condition; there are 15 combinations of this kind.
- 5 flags set: each one of the possible combinations is sufficient to determine the components affected by faults. Hence, they are managed without considering the subsets of three flags; there are 6 possible combinations of this kind.
- All flags set: it is impossible to reconstruct the state from the sensors, so the affected wheel is no longer monitored; there is 1 combination of this kind.

The complete list of the 14 managed combinations, with the indication of the determined components, is reported in Table 3.

#### 5.2.4. Internal Mitigation Algorithms (Traction Subsystem)

We defined two kinds of mitigation strategies:

- Internal: it is possible to isolate this failure at the wheel level without involving a change of the whole rover behavior or/and sensors of other wheels;
- External: it is impossible to manage this failure without involving behavioral changes at the rover level or without using sensors’ external feedback from the affected wheel.

From these two kinds of strategies, we defined four levels of the mitigation algorithm as described in Table 4. The internal ones act at the level of the single wheel without involving the whole rover, while the external ones need measurements or a change of behavior of the entire mobility system.

When one of the 13 failure groups is found, a state request and a mitigation level requirement are generated to trigger the appropriate mitigation.

The states (see Table 5) are numbered in order to have the first ones corresponding to a single failure, and consequently, the double and the triple ones (all encoders failure). In some cases, more than one fault-affected components group is associated with the same state, and this happens for those having the same effects or required mitigation strategies.

There are 3 internal mitigation algorithms capable of mitigating a single failure affecting H-TM, E-TM, and E-TRG:

- H-TM: This uses the best feedback between E-TM, divided by the gear ratio, and E-TRG.
- E-TM: This uses the best feedback between H-TM, divided by the gear ratio, and E-TRG.
- E-TRG: This uses the best feedback between E-TM and H-TM, both divided by gear ratio.

#### 5.2.5. External Mitigation Algorithms (Traction Subsystem)

The external mitigation algorithms are those that also make use of feedback coming from the sensors installed on the other wheels of the rover. Hence, we need to specify both the internal and the external actions. There are five external mitigation algorithms capable of managing failures of the components reported below:

- TRG and TM.
  (Internal) It opens the clutch (TC) to disengage the motor from the wheel.
  (External) The traction control recomputes the torques to the other wheels to consider the impossibility for the affected wheel to generate torque to move the rover.
- TC.
  (Internal) Stops the motor (only to save battery energy) since the TC failure prevents the motor from applying torque on the wheel.
  (External) The same as for TRG and TM.
- H-TM + E-TM or E-TRG + H-TM or H-TM + E-MT (only one of the three).
  (Internal) It uses the still-working speed sensor to feed the rover and motor speed control loop.
  (External) Applies the “inverse kinematic” model of the rover to estimate the speed of the affected wheel to choose the encoder still working correctly and formulate the dual failure diagnosis.
- All encoders.
  (Internal) It configures the motor control routine in the sensorless mode.
  (External) Applies the “inverse kinematic” model of the rover to estimate the speed of the affected wheel to check if the motor is working correctly.
- All encoders + TC and/or + TM and/or TRG.
  (Internal) Stops the motor.
  (External) The traction control recomputes the torques to the other wheels, taking into account the impossibility for the affected wheel to generate torque to move the rover.

#### 5.2.6. Detection Algorithms (Steering Subsystem)

For the steering subsystem, there are three detection algorithms. There are only two encoders, one installed upstream (E-SM) of the SRG, while the other is installed downstream (E-SRG).

- SF1. The flag SF1 is raised when the difference between the angle required for the motor, the position read from the encoder on the motor (E-SM) and the angular position of the motor is greater than 2 rad.
- SF2. The flag SF2 is raised when the difference between the angle measured by the motor (E-SM) encoder is divided by 50, and the angle measured by the encoder on the reduction gear (E-SGR) is greater than the angle measured by the encoder 0.04 rad. (The angle 0.04 rad downstream of the gear is equal to 2 rad upstream of it, taking the gear ratio of the steering system into account, so 2/50 = 0.04).
- SF3. The flag SF3 is raised when the difference between the angle measured by the encoder on the motor (E-SM) and by the encoder on the reduction gear (E-SGR) is greater than 0.04 rad.

Unfortunately, it is possible to trigger a mitigation algorithm only for the cases of the first two rows of Table 6, while in the others, it is not possible since the flags do not allow discrimination between the cases SM, SGR, E-SM + SM, E-SM + SGR, E-SGR + SM, E-SGR + SGR, and E-SM + E-SRG.

#### 5.2.7. Internal Mitigation Algorithms (Steering Subsystem)

There are two internal mitigation algorithms:

- E-SM. This algorithm uses the feedback given by the steering gear reduction encoder E-SGR.
- E-SGR. This algorithm uses the feedback given by steering motor encoder E-SM.

#### 5.2.8. External Mitigations Algorithms (Steering Subsystem)

The absence of the clutch in the steering system collapses the external mitigations only into the rover stop action. To avoid false failure detections, we decided to also check the coherence between the steering angles of the wheels. The failures with external mitigations are:

- SM;
- SGR;
- E-SM + SM;
- E-SM + SGR;
- E-SGR + SM;
- E-SGR + SGR;
- E-SM + E-SGR.

The possibility of checking if the steering system works appropriately is limited only when a steering action is required (the steering angle is controlled in position and not in speed), causing long detection times if the failures affect the steering.

From the mechanical point of view, the most probable failure mode is the SM or the SGR remaining stuck in the last position; hence, the affected wheel remains locked in the last set position.

As it is possible to see in Table 6, SM and SGR raise the same flags. This behavior is expected since their positions are mechanically linked to each other due to the absence of a clutch. Hence, in Table 7, we use the ratio-motor instead of SM and SGR since these two components are not distinguishable.

## 6. Simulation Results

Thanks to the simulation results, it is possible to analyze the software effects on the hardware. In this section, our focus is on determining if the developed mitigation algorithms can mitigate the effects of the failures by comparing the behaviour of the rover with and without them.

Of course, it is possible to determine if the detection algorithms are capable of detecting the failures (and thanks to various scenarios, to determine if, in some cases, there are false positives or negatives) and if the requirements in terms of the failure time tolerance interval (FTTI) [5] are met. We defined some terms as follows:

- Fault time tolerance interval (FTTI) [5]: time-span in which a fault or faults can be present in a system before a hazardous event occurs.
- Fault reaction time interval (FRTI) [5]: time span from the detection of a fault to reaching the safe state.
- Injection time $t_{i}$: the time (from the start of the simulation) in seconds when the considered fault has been injected.
- Detection time $t_{d}$: the time (from the start of the simulation) in seconds when the system has detected the considered fault.
- Diagnostic time interval $t_{dti}$: the difference between the detection time and the injection time, hence $t_{dd}=t_{d}-t_{i}$.
- Mitigation time $t_{m}$: the time when the mitigation algorithm has brought the system to a safe state.
- Mitigation delay $t_{md}$: the difference between the time when the mitigation algorithm has put the system in a safe state and when the fault has been injected, hence $t_{md}=t_{m}-t_{i}$. It is needed that $t_{md}\leq FTTI$.

### 6.1. Achieved Performances

In this subsection, we divided the discussion between the traction and the steering subsystems and, for each one of them, between the two levels of detection (raising of flags and mitigation triggers).

Table 7 and Table 8 report the results obtained from the simulations, respectively, for the traction and the steering subsystem.

### 6.2. Discussion of Results

For each one of the wheels, we found 9 (6 for the central non-steering ones) possible FMs (physical components that can misbehave) and 18 (15 for the central one) combinations of FMs. From the detection point of view, simulations show that 22 of them (75%) are correctly detected, while 5 (25%) are not. These 5 undetected ones are all from the traction subsystem and are those where the TC is involved. In any case, thanks to the simulation results, we found out that these FMs cannot cause critical misbehaviors. The traction clutch (TC) failure makes it impossible to engage it and so to connect the motor to the reduction gear (TRG), hence to the wheel itself. Since the rover still has 5 other running wheels, moves at low speed, and has an advanced traction control system, the loss of the ability of a motor to deliver torque does not make it impossible to keep moving the rover.

There are no false-positive results on the traction subsystem, thanks to the two levels of detection algorithms (Section 5.2.2 and Section 5.2.3) and the coherency checks implicitly provided thanks to the external mitigation algorithms. The same results have also been obtained on the steering subsystem, thanks to the presence of two independent encoders with the adoption of coherency checks due to the rover inverse-Ackerman dynamic model.

Moreover, by the insertion of a virtual sensor (obtained by analyzing the current absorbed), it will be possible to determine the torque provided by the TM to the wheel and, hence, detect the TC failure.

On one hand, the effectiveness of the detection strategies has been described in details due to the simulations results shown in the tables.

On the other hand, the effectiveness of the mitigation strategies, as reported in Section 3.4, has been determined by an informal specification-based analysis: a member of the D.I.A.N.A. team observed the behavior of the rover thanks to the 3D reconstruction offered by CoppeliaSim and accepted all of the mitigation strategies proposed in this paper.

This method is sufficiently objective for the purpose of the design, but is not state-of-the-art. For this reason, we decided to not present the simulation results showing the effectiveness of the mitigation.

## 7. Conclusions

This paper presents a novel simulation-based methodology to aid designers and embedded software developers in improving their reliability analysis of mechatronic systems. It has been applied in a case study about a mobile robotic application. We followed two state-of-the-art manuals, the AIAG&VDA manual for the FMEA and the ECSS-Q-ST-30-02C for the HSIA. We described how to apply it and showed how, thanks to the simulation results, it is possible to obtain objective assessments of severity and detectability in a repeatable way, limited only by the quality of the simulation models.

The introduction has described three advantages of the simulation-based approach over the handmade one. In the first place, it improves the objectivity of the FMEA thanks to the simulation results. These results allow decoupling of the failure effects assessment from the safety engineers’ knowledge. Due to repeating the simulations every time they are needed, it is possible to verify continuously, even from the early development phases, as further subsystems are implemented step by step to the final design if they can fulfill the requirements. In this way, it is possible to check the correctness of the implemented FDIR mechanisms, allowing the developers to verify the detection effectiveness of their embedded software and to verify which subsystems can mitigate their potential failures. Due to the simulation results, it is possible to adopt an iterative approach, modifying the unsatisfactory modules until they can reach the expected performances, allowing them to focus their developing effort on leading to valuable results. In the end, thanks to the possibility of simulating the mechanical components’ behavior and the interaction with the terrain, it proposes a way to simplify the safety assessment (and also in terms of mission requirements) of the hardware/software interactions, especially in those cases where mechanical and electrical/electronic components and the embedded software are involved in determining complex behaviors of the entire robot.

## Figures and Tables

**Table 1:** Failure grouping summary. Here, 0 indicates that the component is fault-free, while 1 indicates that the component failed. The characters “a”, “b” and “x” indicate what components can fail in each group: components marked as “x” can fail simultaneously in any number between 0 and 9; components marked as “a” can fail simultaneously in a maximum number of 2; and components marked as “b” can fail simultaneously in a maximum number of 3 for groups numbered 3, 5, 6, 7, 8 or in a maximum number of 2 for groups numbered 10, 11, 12.

Group number	TM	TRG	TC	H-TM	E-TM	E-TRG	SM	SRG	E-SM	E-SRG	Action at the Rover Level
1	x	x	x	x	x	x	x	1	x	x	Stop.
2	x	x	x	x	x	x	1	x	x	x	Stop.
3	1	0	x	b	b	b	0	0	a	a	Fail (mitigation).
4	x	1	x	x	x	x	x	x	x	x	Stop.
5	x	0	1	b	b	b	0	0	a	a	Fail (rover can work).
6	0	0	0	b	b	b	0	0	1	0	No degradation.
7	0	0	0	b	b	b	0	0	0	1	No degradation.
8	0	0	0	b	b	b	0	0	1	1	Stop.
9	0	0	0	1	1	1	0	0	a	a	Stop.
10	0	0	0	b	b	1	0	0	a	a	No degradation (but check configuration of the rover in order to determine if TRG is failed or not).
11	0	0	0	1	b	b	0	0	a	a	No degradation.
12	0	0	0	b	1	b	0	0	a	a	No degradation.
13	0	0	0	1	1	0	0	0	a	a	No degradation (use as motor feedback the reduction feedback multiplied by 50).

**Table 2:** Flag-failure associations.

	Wheel Velocity Command	E-TM	H-TM	E-TRG
**Wheel velocity command**	-	TF1	TF2	TF6
**E-TM**	TF1	-	TF3	TF4
**H-TM**	TF2	TF3	-	TF5
**E-TRG**	TF6	TF4	TF5	-

**Table 3:** Flag-failure associations for all the components.

Components	TF1	TF2	TF3	TF4	TF5	TF6
**None**	0	0	0	0	0	0
**H-TM**	0	1	1	0	1	0
**E-TM**	1	0	1	1	0	0
**E-TRG**	0	1	1	0	1	0
**TM**	1	1	0	0	0	1
**TRG**	1	1	0	0	0	1
**TC**	0	0	0	1	1	1
**E-TM + H-TM**	1	1	1	1	1	0
**E-TM + E-TRG**	1	0	1	1	1	1
**E-TRG + H-TM**	0	1	1	1	1	1
**E-TM + TC**	1	0	1	1	1	1
**H-TM + TC**	1	0	1	1	1	1
**TM + TC**	1	1	0	1	1	1
**All encoders**	1	1	1	1	1	1

**Table 4:** Traction subsystem mitigation levels.

Legend	Type of Mitigation Algorithm Triggered
0	No mitigation
1.1	Internal mitigation (use another feedback value)
1.2	Internal mitigation (clutch opening)
2.1	External mitigation (use external data, e.g., slip)
2.2	External mitigation (change the behaviour of the rover, e.g., stop the rover)

**Table 5:** Failure-state-mitigation associations for the traction subsystem.

Failure	State	Mitigation Required
None	0	0
H-TM	1	1.1
E-TM	1	1.1
E-TRG	2	2.1
TM	3	1.2
TRG	4	2.2
TC	5	0
E-TM + H-TM	6	1.1
E-TM + E-TRG	6	2.1
E-TRG + H-TM	6	2.1
E-TM + TC	7	0
H-TM + TC	7	0
TM + TC	5	0
All encoders	8	2.1 or 2.2

**Table 6:** Flag-failure associations for the steering subsystem components.

Component	SF1	SF2	SF3
**E-SM**	1	1	0
**E-SGR**	0	1	1
**SM**	1	0	1
**SGR**	1	0	1
**E-SM + SM**	1	0	1
**E-SM + SGR**	1	0	1
**E-SGR + SM**	1	0	1
**E-SGR + SGR**	1	0	1
**Both encoders unreadable; hence, firmware reads 0)**	1	0	1
**Encoders (disagree between each other and with the command)**	1	1	1

**Table 7:** Simulation results for the steering subsystem. Ratio-motor indicates a failure affecting SM or SGR since their positions are mechanically linked.

Injected Failure (s)	Injection Time $t_{i}$ (s)	Detection Time $t_{d}$ (s)	Diagnostic Time Interval (s) $T_{\mathrm{dti}}$ = $t_{i}-t_{d}$	Expected Mitigation Time ($t_{i}+\mathrm{FTTI}$) (s)	Mitigation Time $t_{m}$ (s)	Mitigation Delay $t_{\mathrm{md}}$ (s)	Test Results
Ratio-motor	7.1	12.8 (angle quite constant)	5.7	13.3	15.8	2.5	DETECTED
E-SM	11.6	14.2 (angle quite constant)	2.6	0	0	0	DETECTED
E-SRG	7.5	13.1 (angle quite constant)	5.6	0	0	0	DETECTED
E-SM + E-SGR	8.3 s E-SM + 15.7 s E-SRG	10.6 s E-SM + 17.8 s E-SRG	2.6 s E-SM + 2.1 s E-SRG	16.2	18.6	2.4	DETECTED
E-SM + SM	8.4 s E-SM + 15.8 s SM	11.1 s E-SM + 17.2 s SM	2.7 s E-SM + 1.4 s SM	0 s E-SM+ 17.7 s SM	0 s E-SM + 20 s SM	0 s E-SM +2.3 s SM	DETECTED
E-SRG + SM	6.9 s E-SRG + 12.8 s SM	9.1 s E-SRG + 16 s SM	2.2 s E-SRG + 3.2 s SM	0 s E-SRG + 16.5 s SM	0 s E-SRG + 17.6 s SM	0 s E-SRG+ 1.1 s SM	DETECTED

**Table 8:** Simulation results for the traction subsystem.

Injected Failure (s)	Injection Time $t_{i}$ (s)	Detection Time $t_{d}$ (s)	Diagnostic Time Interval (s) $T_{\mathrm{dti}}$ = $t_{i}-t_{d}$	Expected Mitigation Time ($t_{i}+\mathrm{FTTI}$) (s)	Mitigation Time $t_{m}$ (s)	Mitigation Delay $t_{\mathrm{md}}$ (s)	Test Results
H-TM	11.9	12	0.1	0	0	0	DETECTED
E-TM	10.5	10.7	0.2	0	0	0	DETECTED
E-TRG	8	8.1	0.1	0	0	0	DETECTED
TM	14.6	14.7	0.1	15.2	15.2	0	DETECTED
TRG	13.2	13.9	0.7	14.4	14.4	0	DETECTED
TC	7.7	12.5	4.8	13.5	0	*∞*	FAIL
H-TM + TM	12.4 s H-TM + 17.4 s TM	12.5 s H-TM + 17.5 s TM	0.1 s H-TM + 0.1 s TM	0 s H-TM + 18 s TM	0 s H-TM + 18 s TM	0 s H-TM + 0 s TM	DETECTED
H-TM + TRG	18.6 s H-TM + 20.6 s TRG	18.7 s H-TM + 20.9 s TRG	0.1 s H-TM + 0.3 s TRG	0 s H-TM + 21.4 s TM	0 s H-TM + 21.4 s TRG	0 s H-TM + 0 s TRG	DETECTED
H-TM + TC	5 s H-TM + 13.6 s TC	5.3 s H-TM + 15.2 s TC	0.3 s H-TM + 1.6 s TC	0 s H-TM + 16.2 s TC	0 s H-TM + 0 TC	0 s H-TM + *∞* TC	DETECTED
H-TM + E-TRG	4.7 s H-TM + 22.1 s E-TRG	4.8 s H-TM + 22.2 s E-TRG	0.1 s H-TM + 0.1 s E-TRG	0 s H-TM + 0 s E-TRG	0 s all + 0 E-TRG	0 s H-TM + 0 s E-TRG	FAIL
E-TM + E-TRG	13.8 s E-TM + 21.9 s E-TRG	13.9 s E-TM + 22 s E-TM	0.1 s E-TM + 0.1 s E-TRG	0 s E-TM + 0 s E-TRG	0 s E-TM + 0 s E-TRG	0 s E-TM+ 0 s E-TRG	DETECTED
H-TM + E-TM	9 s H-TM + 15.2 s E-TM	9.1 s H-TM + 15.3 s E-TM	0.1 s H-TM + 0.1 s E-TM	0 s H-TM + 0 s E-TM	0 s H-TM + 0 s E-TM	0 s H-TM + 0 s E-TM	DETECTED
E-TM + TM	7.5 s E-TM + 14.4 s TM	7.6 s E-TM + 14.5 s TM	0.1 s E-TM + 0.1 s TM	0 s E-TM + 15 s TM	0 s E-TM + 15 s TM	0 s E-TM + 0 s TM	DETECTED
E-TM + TC	10.7s E-TM + 17.8 s TC	10.9 s E-TM + 0 TC	0.2 s E-TM + *∞* TC	0 s E-TM + 18.8 s TC	0 s E-TM + 0 s TC	0 s E-TM + *∞* TC	FAIL
E-TM + TRG	6.8 s E-TM + 13.1 s TRG	6.9 s E-TM + 13.8 s TRG	0.1 s E-TM + 0.7 s TRG	0 s E-TM + 14.3 s TRG	0 s E-TM + 14.3 s TRG	0 s E-TM + 0 s TRG	DETECTED
E-TRG + TM	4.5 s E-TRG + 15.3 s TM	4.6 s E-TRG + 15.4 s TM	0.1 s E-TRG + 0.1 s TM	0 s E-TRG + 15.9 s TM	0 s E-TRG + 15.9 s TM	0 s E-TRG + 0 TM	DETECTED
E-TRG + TRG	7.2 s E-TRG +12.6 s TRG	7.3 s E-TRG + 12.7 s TRG	0.1 s E-TRG + 0.1 s TRG	0 s E-TRG + 13.7 s TRG	0 s E-TRG + 13.8 s TRG	0 s E-TRG + 0.1 s TRG	DETECTED
E-TRG + TC	8.7 s E-TRG + 15 s TC	8.8 s E-TRG + 0 s TC	0.1 s E-TRG + *∞* TC	0 E-TRG + 16 s TC	0 s E-TRG + 0 s TC	0 s E-TRG + *∞* TC	FAIL
TM + TC	7.9 s TM + 13.1 s TC	8 s TM + 13.2 s TC	0.1 s TM + 0.1 s TC	8.5 s TM + 14.2 s TC	8.5 s TM + 0 s TC	0 s TM + *∞* TC	DETECTED
TC + TRG	2.4 s TC + 15.7 s TRG	2.4 s TC + 15.9 s TRG	0 s TC + 0.2 s TRG	3.4 s TC + 16.4 s TRG	15.9 s TC + 16.9 s TRG	12.5 s TC + 0.5 s TRG	FAIL
TRG + TC	13.9 s TRG + 15.2 s TC	14.6 s TRG + 16.8 s TC	0.7 s TRG + 1.6 s TC	15.1 s TRG + 17.8 s TC	15.1 s TRG + 0 s TC	0 s TRG + *∞* TC (not needed)	DETECTED
All sensors	10.8 s H-TM + 12.9 s E-TM+ 15.4 s E-TRG	10.9 s H-TM + 13 s E-TM + 15.4 s E-TRG	0.1 s H-TM + 0.1 s E-TM + 0 s E-TRG	0 H-TM + 0 E-TM + 16.4 s E-TRG	0 H-TM + 0 E-TM + 17.6 s E-TRG	0 H-TM + 0 E-TM + 1.2 s E-TRG	DETECTED

## Data Availability

Not applicable.

## Abbreviations

The following abbreviations are used in this manuscript:

$t_{dti}$	Diagnostic time interval
$t_{md}$	Mitigation delay
$t_{d}$	Detection time
$t_{i}$	Injection time
$t_{m}$	Mitigation time
AIAG	Automotive Industry Action Group
AP	Action priority
API	Application program interface
BLDC	Brushless direct current
CAD	Computer-aided design
COTS	Commercial off-the-shelf
CSV	Comma separated values (file)
ECU	Electronic control unit
E-SM	Absolute encoder installed to measure the position of the SM
E-SRG	Absolute encoder installed on the steering after the SRG
E-TM	Relative encoder installed on the TM
E-TRG	Relative encoder installed on the TRG
FC	Failure cause
FDIR	Fault detection, isolation, and recovery
FM	Failure mode
FMEA	Failure mode and effect analysis
FMEDA	Failure mode, effect and diagnostic analysis
FRTI	Fault reaction time interval
FTTI	Fault tolerance time interval
HARA	Hazard analysis and risk assessment
HSIA	Hardware-software interaction analysis
H-TRG	Hall effect speed sensor installed on the TRG
MQTT	Message queue telemetry transport
RHF	Random hardware faults
SF	Steering flag
SM	Steering stepper motor
SRG	Steering reduction gear
TC	Clutch between the TM and TRG
TF	Traction flag
TM	Traction motor
TRG	Traction reduction gear
VDA	German Association of the Automotive Industry
